# Treatment outcome and compliance to dose-intensified linac-based SBRT for unfavorable prostate tumors using a novel real-time organ-motion tracking

**DOI:** 10.1186/s13014-021-01908-0

**Published:** 2021-09-17

**Authors:** Raffaella Lucchini, Denis Panizza, Riccardo Ray Colciago, Veronica Vernier, Martina Camilla Daniotti, Valeria Faccenda, Stefano Arcangeli

**Affiliations:** 1 Department of Radiation Oncology, School of Medicine and Surgery, University of Milan Bicocca, Milan, Italy; 2 Radiation Oncology Department, ASST Monza, Monza, Italy; 3 Medical Physics Department, ASST Monza, Monza, Italy; 4 Department of Physics, University of Milan Bicocca, Milan, Italy; 5 Department of Physics, University of Milan, Milan, Italy

**Keywords:** Prostate cancer, SBRT, Organ-motion tracking

## Abstract

**Purpose/objectives:**

To report preliminary data on treatment outcome and compliance to dose-intensified organ sparing SBRT for prostate cancer using a novel electromagnetic transmitter-based tracking system (RayPilotÒ System) to account for intra-fractional organ motion.

**Material/methods:**

Thirteen patients with intermediate unfavorable (9) and selected high-risk (4) prostate cancer underwent dose-escalated SBRT in 4 or 5 fractions (BED_1.5_ = 279 Gy and 253 Gy, respectively). The VMAT treatment consisted in two 6FFF or 10FFF full arcs optimized to have the 95% isodose covering at least 95% of the PTV (2 mm isotropic expansion of the CTV). Whenever the real-time tracking registered a displacement that exceeded 2 mm during the setup and/or the beam delivery, the treatment was interrupted and the prostate motion was promptly corrected. The incidence of treatment-related genitourinary (GU) and gastrointestinal (GI) toxicity, patient QoL and PSA outcomes were computed from the start of treatment to the last follow-up date.

**Results:**

All patients completed the treatment in the expected time (10.2 +/− 4.2 minutes) and their compliance to the procedure was excellent. No clinically significant acute Grade 2 or higher GI (rectal) and GU side effects were observed within 90 days from the treatment completion. The median IPSS increased from 8 at baseline to 12 one-month after treatment and settled to 6 at 3 months. EPIC-26 scores in the urinary domain decreased from a median baseline of 86 pre-treatment to 79 at one-month and returned to baseline at a later timepoint (median score of 85 at 3 months). EPIC-26 scores in the bowel domains did not show significant changes within 3 months following RT. The prostate was found within 1 mm from its initial position in 78% of the beam-on time, between 1 and 2 mm in 20%, and exceeded 2 mm only in 2%, after correction for motion which was performed in 45% of the fractions, either during setup or beam delivery.

**Conclusions:**

Our preliminary findings show that dose intensified SBRT for unfavorable prostate tumors does not come at the cost of an increased toxicity, provided that a reliable technique for real time prostate monitoring is ensured. Fast FFF beams contributed to reduce intra-fractional motion. These observations need to be confirmed on a larger scale and a longer follow up.

## Background

Conventional dose-escalated radiation therapy (RT) for organ-confined prostate cancer involves the delivery of a single 1.8–2.0 Gy fraction, five days per week, for eight-nine weeks to a total dose of 76–80 Gy. This regimen is based on four randomized trials and a metanalysis [1–5] showing improved progression-free free survival compared to lower cumulative doses, but at the cost of increased toxicity which prevents further dose escalation. Given this concomitant increase in toxicity with dose, as well as the expense and inconvenience of such a protracted course, alternative treatment schedules have been investigated. Clinical results from retrospective studies have led to the hypothesis that the α/β of prostate cancer is lower than that of the majority of human tumours, close to a value that is characteristic of late responding tissues [6–8]. Based on this assumption, the delivery of fewer and larger fractions (hypofractionation) than used in conventional RT, might effectively improve the therapeutic ratio while maintaining isoeffective tumour doses, and shortening overall treatment time.

This has inspired a number of clinical trials assessing the optimal dose per fraction when treating prostate cancer, and some of them have demonstrated the non-inferiority of moderate hypofractionation (eg, 20 treatments) to conventional RT [9–12] in terms of efficacy and toxicity.

Along with tremendous advances in radiation technology that have enabled improved precision in the beam delivery, shorter radiation schedules than previously possible can now be implemented without compromising treatment efficacy, thus increasing patients’ compliance and the cost-effectiveness profile of RT. Stereotactic body radiation therapy (SBRT) is characterized by the use of a high radiation dose per delivered fraction through highly intensity-modulated beams, generating sharper dose fall-off and enhanced dose conformity to the target, which is ensured by strict adherence to the planned treatment via daily imaging. So far, most of the supporting evidence in favour of SBRT comes from two large systematic reviews [13, 14] and the results of one phase III study, HYPO-RT-PC [15]. Indeed, an American Society for Radiation Oncology/American Society of Clinical Oncology/American Urological Association (ASTRO/ASCO/AUA) guideline included recommendations regarding the use of ultrahypofractionation (eg, SBRT) in the treatment of low-intermediate risk prostate cancer [16]. Evidence has accumulated that SBRT for patients with low and intermediate risk prostate cancer is associated with excellent biochemical outcomes and acceptably low toxicity rates [17]. However, caution is advised when dose-escalated SBRT aimed at maximizing tumor control for more aggressive disease is needed, in view of the non-negligible risk of high grade toxicity [18]. The aim of the present study is to report preliminary data on treatment outcome and compliance to dose-intensified organ-sparing SBRT for intermediate and selected high-risk prostate cancer using a novel electromagnetic transmitter-based tracking system to account for intra-fractional organ motion.

## Methods

### Patients

Patients over the age 50 with histologically confirmed organ-confined prostate adenocarcinoma considered at intermediate unfavorable and selected high risk (eg. Gleason Grade Group V, cT3b disease and prostate specific antigen (PSA) > 20 ng/mL excluded) as per National Comprehensive Cancer Network definition, with an international prostate symptoms score (IPSS) ≤ 19 (alpha-blockers allowed) and a compute tomography (CT), magnetic resonance imaging (MRI) or Ultrasound-based volume estimation of prostate gland ≤ 100 g were included. All patients, but 4 (who refused any form of endocrine manipulation), received concomitant androgen deprivation therapy (ADT) as per standard of care [19]. Institutional review board approval was obtained, and all participants provided written consent.

### Treatment planning and radiation delivery

**Fig. 1:**
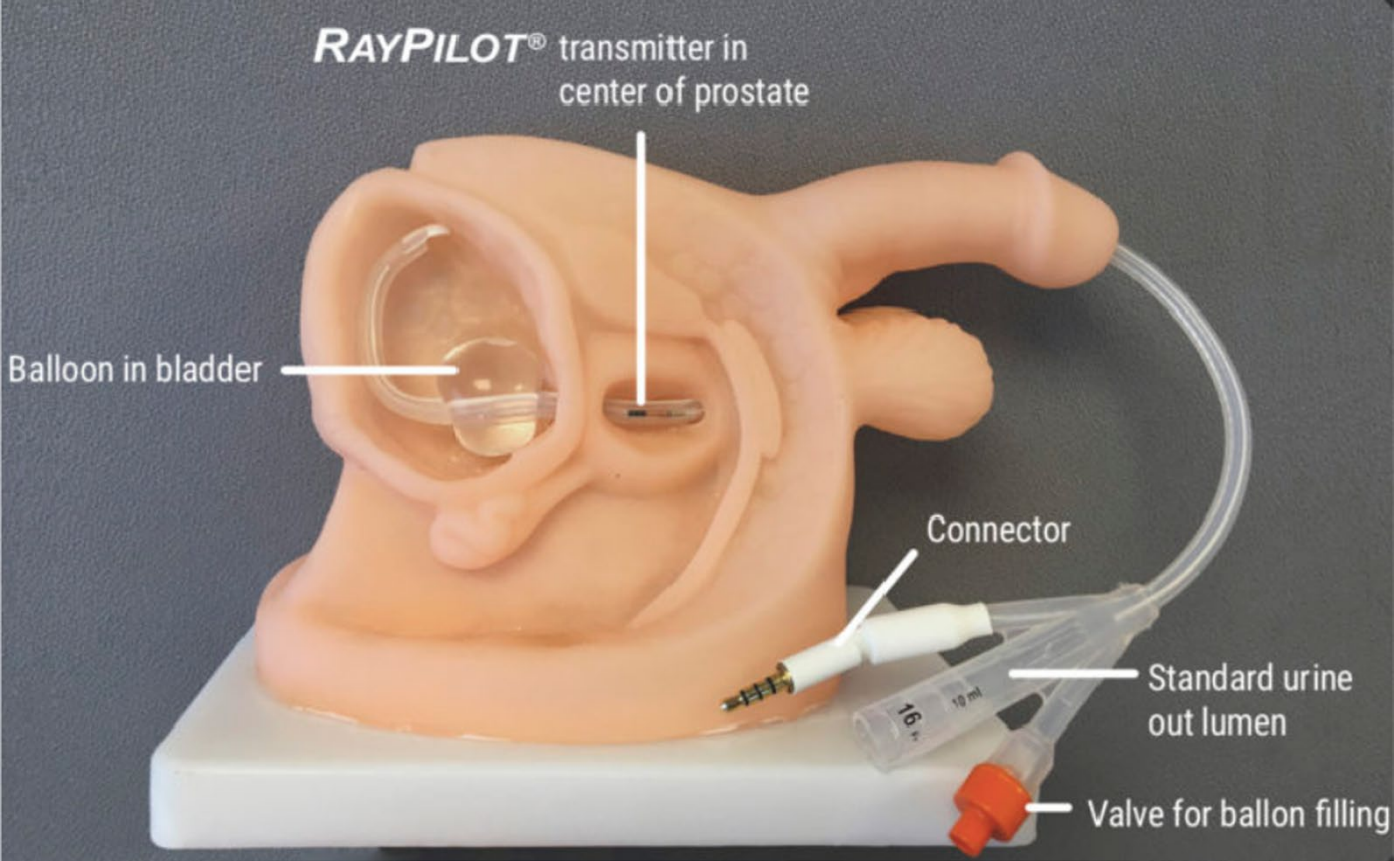
RayPilot main components

Patients were immobilized in supine position using FeetFix® (CIVCO Medical Solutions, Iowa, US) system anchored to the couch for ankle fixation, with arms placed over their chest. A micro-enema was administered before simulation and each treatment to assess anatomical reproducibility. A monitoring system (RayPilot® System by Micropos Medical AB, Gothenburg, Sweden) provided real-time localization of the prostate based on electro-magnetic detection of a transmitter, which was placed intra-urethrally by means of a dedicated catheter to identify anatomy and allow intra-fractional tracking (Fig. 1). The same catheter was used to fill the bladder with 100 cc of saline solution.

A T2W MRI was acquired in treatment position and fused with the simulation CT to accurately delineate the target volume and the organs-at-risk. The clinical target volume (CTV) was the prostate and the seminal vesicles. The planning target volume (PTV) included CTV with a 2 mm isotropic 3D-margin. A margin of 2 mm was applied around the catheter to determine a planning organ at risk volume (PRV) for the urethra in order to provide a significant dose-sparing at this level, allowing a negative dose-painting to reduce the risk of treatment-related urinary toxicity. A Volumetric Modulated Arc Therapy treatment consisted in two 6 MV or 10 MV flattening filter free (FFF) full arcs optimized to have the 95% isodose covering at least 95% of the PTV. SBRT was scheduled every day for a total dose either of 40 Gy in 5 fractions or 38 Gy in 4 fractions. The corresponding Biologically Effective Dose considering an α/β ratio of 1.5 Gy was 253 Gy and 279 Gy, respectively.

Mandatory dose-volume constraints were defined for both target coverage and avoidance of normal adjacent tissues, including rectum, rectum wall, bladder, PRV of urethra and penile bulb, as shown in Table 1. Accurate patient setup was obtained by ConeBeam-CT (CBCT) before treatment, and real-time motion tracking ensured that both the setup and the beam delivery phases were interrupted and corrected whenever the displacement exceeded a predetermined 2 mm threshold. In case of prolonged drift outside this tolerance, a new CBCT was prompted and matched to correct for prostate motion.

**Table 1:** Planning objectives for dose-escalated prostate SBRT

	Dose objective	Expected (4 fractions)	Expected (5 fractions)	Priority
Rectum	Dmax (0.035 cc)	38 Gy (100% prescribed dose)	42 Gy (105% prescribed dose)	1
	D5%	≤ 33 Gy	≤ 40 Gy	2
	D10%	≤ 29 Gy	≤ 36 Gy	2
	D20%	≤ 26.5 Gy	≤ 32 Gy	2
	D50%	≤ 16.7 Gy	≤ 20 Gy	2
Rectal mucosa	Dmax (0.035 cc)	28.5 (75% prescribed dose)	–	1
Bladder	Dmax (0.035 cc)	45.6 Gy (120% prescribed dose)	44 Gy (110% prescribed dose)	1
	D10%	≤ 41.8 Gy (110% prescribed dose)	≤ 38 Gy (95% prescribed dose)	1
	D40%	≤ 16.6 Gy	≤ 20 Gy	2
PRV Urethra	Dmax (0.035 cc)	45.6 Gy (120% prescribed dose)	48 Gy (120% prescribed dose)	1
	D10%	≤ 41.8 Gy (110% prescribed dose)	≤ 44 Gy (110% prescribed dose)	1
Penile bulb	Dmax (0.035 cc)	38 Gy (100% prescribed dose)	40 Gy (100% prescribed dose)	3

### Toxicity and quality of life assessment

Toxicity, as defined by National Cancer Institute Common Terminology Criteria for Adverse Events v.5.0, was assessed during treatment, at one-month and at 3 months. IPSS [20] and Expanded Prostate Cancer Index Composite Short Form (EPIC-26) bowel and urinary Quality of Life (QoL) [21] scores were collected once prior to treatment and then following treatment at the above time points via questionnaries. The incidence of acute treatment related genitourinary (GU) and gastrointestinal (GI) toxicity, patient QoL and PSA outcomes were computed from the start of treatment to the last follow-up date.

## Results

### Patient characteristics

From June 2020 to May 2021, 13 patients were included. Median age was 77 years (range 63–81). Intermediate unfavorable and high-risk prostate cancer accounted for 69% and 31% respectively. Median PSA at baseline was 9.78 ng/mL (range 4.99–20). Median CTV and PTV were 47.05 cc (range 32.06–96.71) and 66.6 cc (range 48.89–128.53), respectively. Patients, tumors and treatment characteristics are summarized in Table 2.

**Table 2:** Baseline patients, tumors and treatment characteristics

Age		
Median	77	Range [63–81]
Comorbidities		
None	4 (30.7%)	
1 Comorbidity	4 (30.7%)	
> 1 Comorbidities	5 (38.6%)	
Anticoagulants		
Yes	5 (38.6%)	
No	8 (61.4%)	
Alpha blockers		
Yes	5 (38.6%)	
No	8 (61.4%)	
IPSS		
Median	8	Range [2–14]
Prostate volume (mL)		
Median	35.5	Range [24–80]
NCCN risk group		
Intermediate unfavorable	7 (69.2%)	
High	3 (23.1%)	
Very High	1 (7.7%)	
Gleason score		
7 (4 + 3)	9 (69.2%)	
8 (4 + 4)	2 (15.4%)	
9 (4 + 5)	1 (7.7%)	
10 (5 + 5)	1 (7.7%)	
ISUP grading group		
3	9 (69.2%)	
4	2 (15.4%)	
5	2 (15.4%)	
Clinical stage		
T2a	4 (30.8%)	
T2b	2 (15.4%)	
T2c	6 (46.1%)	
T3a	1 (7.7%)	
PSA level (mg/mL)		
Median	9.78	Range [4.99–20]
< 10	8 (61.4%)	
10–20	5 (38.6%)	
Radiation therapy prescribed and delivered		
40 Gy in 5 fractions	4 (30.8%)	
38 Gy in 4 fractions	9 (69.2%)	
CTV (cc)		
Median	47.05	Range [32.06–96.71]
PTV (cc)		
Median	66.60	Range [48.89–128.53]
PTV (D95)		
Median	96%	Range [95–97%]

### Organ motion mitigation

In 56 treated fractions, 86 CBCT to planning CT matchings were performed. In 31/56 fractions (55%), the signal was within the 2 mm threshold for the whole time. Interruption triggered by the tracking system occurred in 25/56 (45%) of the monitored fractions and a new CBCT was mandated. Specifically, in 15 fractions (27%), at least one CBCT was repeated during the initial setup phase before starting the beam delivery. In 10 fractions (18%), the treatment was interrupted and the patients were repositioned. Mean delivery time (beam-on time ± interruptions) was 3.5 ± 0.9 min (2.5–7.3), mean time to treatment from patient setup to beam-off was 10.2 ± 4.2 min with a median time of 8 min (5.5–22.7). The mean value of the target average deviation was − 0.18 mm, 0.01 mm, and − 0.26 mm in lateral, longitudinal, and vertical direction, respectively, indicating a negligible systematic component (Table 3). All data points from the 56 analyzed fractions were used to evaluate the percentage of time that the transmitter (and thus the prostate) was offset from its reference position. For the purposes of histogram analysis, displacement from the reference position was divided into 1-mm increments. The prostate was found within 1 mm from its initial position in 78% of the delivery time, between 1 and 2 mm in 20%, and exceeded 2 mm only in 2%. When considering the overall treatment time (setup time + beam-on time + interruptions), the same features were 83% (within 1 mm), 13% (between 1 and 2 mm), and 4% (in excess of 2 mm), respectively.

**Table 3:** Summary of trajectory evaluation from intrafractional prostate monitoring: mean, standard deviation and maximum displacement values

Treatment phase	Displacement (mm)	Directions		
		Lateral	Longitudinal	Vertical
Setup	Mean	− 0.20 ± 0.45	− 0.07 ± 0.74	− 0.21 ± 0.81
	Maximum	4.88	7.75	17.73
Dose delivery	Mean	− 0.14 ± 0.41	0.15 ± 0.70	− 0.33 ± 0.73
	Maximum	3.09	5.23	12.74
Global treatment	Mean	− 0.18 ± 0.46	0.01 ± 0.77	− 0.26 ± 0.82
	Maximum	4.88	6.20	17.73

### Treatment outcome

All patients completed the treatment in the expected time and their compliance to the procedure was excellent. No clinically significant acute Grade 2 or higher GI (rectal) and GU side effects were observed within 90 days from the treatment completion. At 30-days, only one (7.7%) patient experienced acute Grade 1 GI toxicity (proctitis), while acute Grade 1 GU toxicity (dysuria) occurred in five (38.6%) patients. At 90-days, Grade 1 GI and Grade 1 GU toxicity occurred in two (15.4%) and five (38.6%) patients, respectively (Table 4). At 3 months, a PSA assessment showed a median value of 1.85 ng/mL (range 0.01–3.86 ng/mL).

**Table 4:** Rates of 30 days and 90 days side effects from the start of treatment

Genitourinary toxicity			Gastrointestinal toxicity	
	30 days	90 days	30 days	90 days
*Grade*				
1	5 (38.6%)	5 (38.6%)	1 (7.7%)	2 (15.4%)
2	0 (0)	0 (0)	0 (0)	0 (0)
≥ 3	0 (0)	0 (0)	0 (0)	0 (0)

### Quality of life

**Fig. 2:**
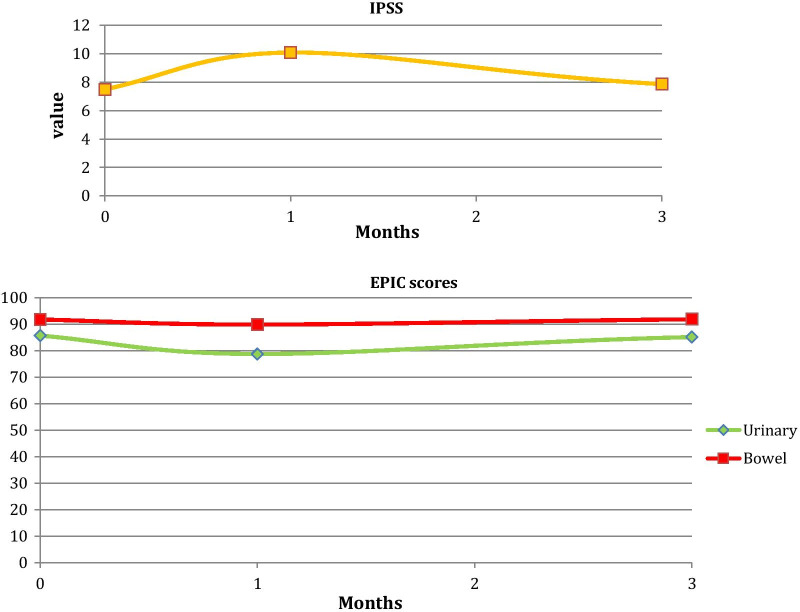
Time course of IPSS and EPIC-26 scores for bowel and urinary domains

The median IPSS increased from 8 at baseline to 12 one-month after treatment, and settled at 6 at 3 months, approximating the pre-treatment baseline value. Consistent with the results of the IPSS, EPIC-26 scores in the urinary domain decreased from a median baseline of 86 pre-treatment to 79 at one-month and returned to baseline at a later timepoint (median score of 85 at 3 months). There was no significant decrease in the 3 months median EPIC-26 scores in the bowel domains. (Fig. 2).

## Discussion

Mounting evidence show favorable outcomes for low- and intermediate-risk prostate cancer patients treated with SBRT with short- and medium-term follow-up [22–41], and the ASTRO/ASCO/AUA guideline has recently issued a shared guideline lending support to the use of SBRT for prostate cancer, which has matured to a point where it can be considered an appropriate alternative to both conventional and moderately hypofractionated RT [16]. The most popular schedule is 35–36.25 Gy in five fractions, which carried excellent results, with a 5-year biochemical-Disease Free Survival (b-DFS) ranging from 94 to 97% in low-risk patients, but appears to be suboptimal in intermediate risk patients, who experienced a 5-year b-DFS of only 84%, as showed in a pooled analysis of eight institutions [42]. The attempt to further escalated the dose, however, was associated with unacceptable toxicity: in a dose-escalation trial [18], 6.6% of patients treated at the highest dose level (50 Gy in five fractions) developed high grade rectal toxicity, 5 of whom required colostomy. In addition, the odds of having a late grade 2 + GU toxicity were 18-fold higher for patients treated with SBRT schedules of 40 Gy compared to those treated with 35 Gy [36]. Not even the use of proton seems to hold sufficient promises for SBRT dose intensification, in view of the disappointing results of two normo-fractionated trials, showing a 50% greater incidence of rectal toxicity compared to IMRT [43], and a significant (8%) late grade 3 rectal toxicity when proton dose was escalated to 82 Gy [44], respectively. Additionally, since only preliminary data of a randomized phase II trial comparing different SBRT schedules for favorable risk prostate cancer have been published [45], the optimal dose for prostate SBRT is yet to be defined [46]. In this report of dose-intensified organ-sparing SBRT for unfavorable risk prostate cancer, low rates of genitourinary and gastrointestinal toxicity were observed with little change in QoL by IPSS and EPIC-26 scores. Despite the high dose employed in this series, the early toxicity outcomes are in keeping with the large majority of SBRT trials, where lower doses have been used, and definitely better when compared with dose-escalated regimens (Table 5). The low-toxicity profile in this study may be attributed to the strict adherence to planning criteria and the application of tight margins around the CTV, as well as the restrictive selection criteria, including the selection of patients with prostate volume ≤ 100 g and IPSS scores < 19. While without continuous monitoring and intervention, in approximately 10% of patients intra-fractional motion would lead to target missing [47], the use of a novel electromagnetic transmitter-based tracking system afforded sub-millimeter precision in tumor targeting during treatment delivery, enabling the reduction of safety margins up to 2 mm. Furthermore, the location of the urethra was clearly identified on the fused CT/MRI set by virtue of the catheter. With a 2 mm expansion around the catheter, negative dose-painting around the urethra significantly contributed to reduce the risk of GU toxicity. Ultimately, the very high dose rates available with the use of FFF beams allowed a significant reduction of total session treatment time, thus decreasing the risk of intra-fraction motion, which might have resulted in less toxicity and accordingly in a better QoL. Similar to the mild toxicity in this study, follow-up extending out to 6 months post-treatment showed limited to no change in QoL as measured by either IPSS, or EPIC-26 scores in both the urinary and bowel domains. Mean EPIC urinary and bowel QoL declined at one-month post-treatment, but almost settled to baseline by 3 months. Our findings should be interpreted with caution given the low rate of events and the short follow-up that cannot capture long-term adverse effects, nor meaningful differences in treatment outcomes compared to similar SBRT regimens. Likewise, the ability to draw any conclusion on the efficacy of high-dose SBRT is scarce. However, as the rate and magnitude of PSA decline following definitive RT for prostate cancer seem to be correlated with clinical outcomes [48–50], the median value of PSA nadir at 3 months in our series indicates an optimal treatment response at least at a very early timepoint. Nevertheless, our study shows that, unlike dose-escalation experiences either with standard fractionated External Beam RT [1–4], protontherapy [44] or SBRT [18] for prostate cancer, the worthy cost-effectiveness profile of our approach does not necessarily come at the cost of an increased toxicity, provided that a close attention is paid to ensure pelvic anatomy reproducibility and target stability during treatment. While the results of this study are hypothesis generating, their validation on a larger scale is needed to implement strategies for safe dose escalation in the SBRT setting based on novel techniques that can reduce intrafractional prostate motion.

**Table 5:** Previously published rates of toxicity following prostate SBRT

Study	n	Dose/fractions	Scale	Genitourinary toxicity		Gastrointestinal toxicity	
				Grade 2	≥ Grade 3	Grade 2	≥ Grade 3
Kim [16]	91	45–50/5	CTCAE v.3	Acute 22% Late 20.9%	Acute 0% Late 5.5%	Acute 20.9% Late 13.2%	Acute 2.2% Late 6.6%
Madsen [21]	40	33.5/5	CTCAE v.2	Acute 20.5% Late 20%	Acute 2.5% Late 0%	Acute 13% Late 7.5%	Acute 0% Late %
Tang [22]	30	35/5		Acute 13%	Acute 0%	Acute 7%	Acute 0%
McBride [23]	34	37.5–36.25/5	CTCAE v.4	Acute 19% Late 17%	Acute 0% Late 2%	Acute 7% Late 7%	Acute 0% Late 5%
Alongi [24]	40	35/5	CTCAE v.4	Acute 40% Late 2.5%	Acute 0% Late 0%	Acute 10% Late 0%	Acute 0% Late 0%
Boyer [25]	60	37/5	CTCAE v.4	Late 6.7%	Late 0%	Late 8.3%	Late 1.7%
King [26]	67	36.25/5	RTOG	Late 5%	Late 3.5%	Late 2%	Late 0%
Bolzicco [27]	100	35/5	RTOG	Late 3% Acute 12%	Late 1% Acute 0%	Late 1% Acute 18%	Late 0%
Elias [28]	84	35/5	RTOG	Late 5.9% Acute 20.2%	Late 0%	Late 7.1% Acute 9.5%	Late 1.1%
Katz [29]	515	35–36.25/5	RTOG	Late 9.1% Acute 4%	Late 1.7% Acute 0%	Late 4% Acute 4%	Late 4% Acute 0%
Bernetich [30]	142	35–36.25–37.5/5	CTCAE v.3	Late 14% Acute 28%	Late 2% Acute 2%	Late 3% Acute 4%	Late 0% Acute 0%
Gurka [31]	208	35–36.25/5	CTCAE v.4	Late 2.4% Acute 0.9%	Late 1.4% Acute 0%	N.A	N.A
Seymour [32]	56	38/4	CTCAE v.4	Late 19.6% Acute 35.7%	Late 3.6% Acute 0%	N.A	N.A
Qi [33]	86	40/5	EPIC QoL	Obs/irrit MID: 46% Incont MID: 28%	N.A	N.A	N.A
Kole [34]	216	35–36.25/5	IPSS	Late 13%	N.A	N.A	N.A
Helou [35]	259	35–40/5	RTOG	Late 32.6%	Late 1.9%	Late 12.9%	Late 1.1%
Zhang [36]	78	38/4	CTCAE v.4	Late 19.2%	Late 2.6%		
Jackson [37]	66	37/5	CTCAE v.4	Late 1.9% Acute 23%	Late 0% Acute 0%	Late 5% Acute 4%	Late 0% Acute 0%
Musunuru [38]	258	35–40/5	CTCAE v.3	N.A	N.A	Late 16.2%	Late 3.2%
Miszczyk [39]	400	36.25/5	RTOG	Late 2.9% Acute 4%	Late 0% Acute 0.4%	Late 0.6% Acute 1.6%	Late 0.3% Acute 0.4%
Zelefsky [40]	551	35–40/5		Late 21.1% Acute 10%	Late 2.5% Acute 0.7%	Late 3.4% Acute 1.8%	Late 0.4% Acute 0%
Current series	15	38/4 40/5	CTCAE v.5	Acute 0% Late 0%	Acute 0% Late 0%	Acute 0% Late 0%	Acute 0% Late 0%

## Data Availability

The datasets supporting the conclusions of this article are included within the article.

## Abbreviations

RT: Radiation therapy
SBRT: Stereotactic body radiation therapy
ASTRO: American Society for Radiation Oncology
ASCO: American Society of Clinical Oncology
AUA: American Urological Association
PSA: Prostatic specific antigen
IPSS: International prostatic symptoms score
CT: Computed tomography
ADT: Androgen deprivation therapy
MRI: Magnetic resonance image
FFF: Flattening filter free
PTV: Planning target volume
CTV: Clinical target volume
PRV: Planning organ at risk volume
GU: GenitoUrinary
CBCT: Cone beam computed tomography
EPIC: Expanded prostate cancer index composite short form
QoL: Quality of life
GI: GastroIntestinal
b-DFS: Biochemical-Disease free survival
